# Treatment of Hyperpigmentation After Sclerotherapy Through Mesotherapy With Deferoxamine Mesylate: A Case Series

**DOI:** 10.7759/cureus.98954

**Published:** 2025-12-11

**Authors:** Brenno Augusto S Mello Netto, Eduardo Zeilmann, Gilmar S Santos, José Marcelo Corassa

**Affiliations:** 1 Vascular Surgery, Seabra Excelencia Vascular, Vitória, BRA; 2 Vascular Surgery, Instituto de Flebologia Moderna (IFE), Itajaí, BRA; 3 Vascular Surgery, Vein Clinic, Salvador, BRA; 4 Vascular Surgery, Santa Paula Hospital, Vitória, BRA

**Keywords:** cutaneous hyperpigmentation, esclerotherapy, hemosiderin deposition, mesotherapy, post-inflammatory hyperpigmentation, varicose veins

## Abstract

Post-sclerotherapy hyperpigmentation is a relatively frequent complication, often associated with considerable aesthetic concern and potential long-term persistence. The underlying pathophysiology mainly involves dermal hemosiderin deposition, inflammatory responses, and secondary melanogenesis induced by the local inflammatory environment. Deferoxamine mesylate, a well-established iron-chelating agent, has attracted attention as a potential therapeutic modality for hyperpigmentation, aiming to improve clinical outcomes. This case series presents the therapeutic experience of intradermal administration (mesotherapy) of deferoxamine mesylate in patients with persistent post-sclerotherapy hyperpigmentation.

This case series describes three female patients aged 35, 58, and 45 years, respectively, who presented with persistent hyperpigmentation lasting more than three months after sclerotherapy. All patients underwent monthly intradermal mesotherapy using deferoxamine mesylate. Treatment response, safety, and patient satisfaction were assessed.

Two patients achieved satisfactory pigmentation lightening after three sessions, while only one patient required four sessions to achieve adequate lightening of the affected skin. All participants reported high levels of satisfaction with the aesthetic result. No significant adverse effects were documented, except for mild (erythema) and transient local reactions at the injection sites.

Mesotherapy with deferoxamine mesylate demonstrated a favorable safety profile and promising efficacy in the treatment of persistent hyperpigmentation after sclerotherapy. These findings corroborate its potential for incorporation as an adjuvant therapeutic option in the clinical arsenal for pigmentation disorders following vascular procedures.

## Introduction

Skin hyperpigmentation is a relatively frequent adverse consequence after vascular interventions, such as sclerotherapy, transdermal or endovenous laser therapy, and varicose vein surgery. Its incidence has been reported in up to 30% of cases after sclerotherapy, varying according to the procedure technique, the type of sclerosing agent, the patient's phototype, and post-treatment care [1,2].

The underlying pathophysiology is multifactorial, involving two main mechanisms: (1) dermal hemosiderin deposition secondary to erythrocyte extravasation and hemolysis, resulting in iron release into surrounding tissues [3]; and (2) melanocytic stimulation induced by localized inflammatory processes, leading to melanin deposition in the epidermis and/or dermis [4,5].

Although spontaneous regression can occur, resolution is usually prolonged, ranging from several months to years, and can impose a significant aesthetic and psychosocial burden on affected individuals [6]. Current therapeutic modalities, including topical depigmenting agents (e.g., hydroquinone, kojic acid, and retinoic acid), chemical peels, microneedling, and various laser systems, present inconsistent and often suboptimal results [7].

Deferoxamine mesylate, an iron-chelating agent traditionally used in systemic disorders such as hemochromatosis and transfusion-related hemosiderosis, has been investigated for localized subcutaneous administration. In a pioneering clinical study, Lopez et al. (2001) demonstrated a marked acceleration in the elimination of post-sclerotherapy hyperpigmentation in patients treated with deferoxamine (27-46 days) compared to the natural resolution period (150-255 days) [8]. Recent histopathological investigations have further revealed the coexistence of hemosiderin and melanin deposits in post-sclerotherapy pigmentation, reinforcing the need for therapeutic approaches that address both pathogenic pathways [3].

However, the aforementioned study promoted subcutaneous injection of the medication, and pigment deposition is more frequent in the dermis. Thus, we deduced that mesotherapy could allow for targeted intradermal administration of deferoxamine mesylate directly into the affected tissue, representing an innovative and potentially safe therapeutic strategy. This study describes a case series of three patients with post-sclerotherapy hyperpigmentation treated with intradermal deferoxamine mesylate, evaluating the efficacy of this technique and this medication for lightening pigmentation, possible adverse effects, and the time required for pigmentation resolution.

This case series presents three consecutively treated patients with persistent post-sclerotherapy hyperpigmentation who underwent intradermal deferoxamine mesylate. We evaluated treatment efficacy, safety, and time to clinical improvement.

## Case presentation

Three female patients (mean age: 46 years; range: 35-58 years) with persistent hyperpigmentation for more than three months after sclerotherapy with polidocanol or hypertonic glucose were selected for this study. Fitzpatrick skin phototypes ranged from III to IV. All patients provided informed consent before treatment and photographic documentation.

Three consecutively treated female patients (mean age: 46 years; range: 35-58 years) presented with persistent hyperpigmentation following sclerotherapy and were managed with monthly intradermal mesotherapy using deferoxamine mesylate. The medication (vial containing 500 mg lyophilized powder) was diluted only with the standard diluent provided by the drug manufacturer to a final concentration of 50 mg/mL. Intradermal therapy was performed using a 30-gauge needle, administering 0.1-0.2 mL per injection point, spaced approximately 1 cm apart, only in the affected area. The treatment endpoint was the patient's perception of improvement and satisfaction.

All underwent standardized photographic documentation and clinical evaluation (including assessment of possible adverse events) during follow-up. The individual courses are described below.

Case one

**Figure 1:**
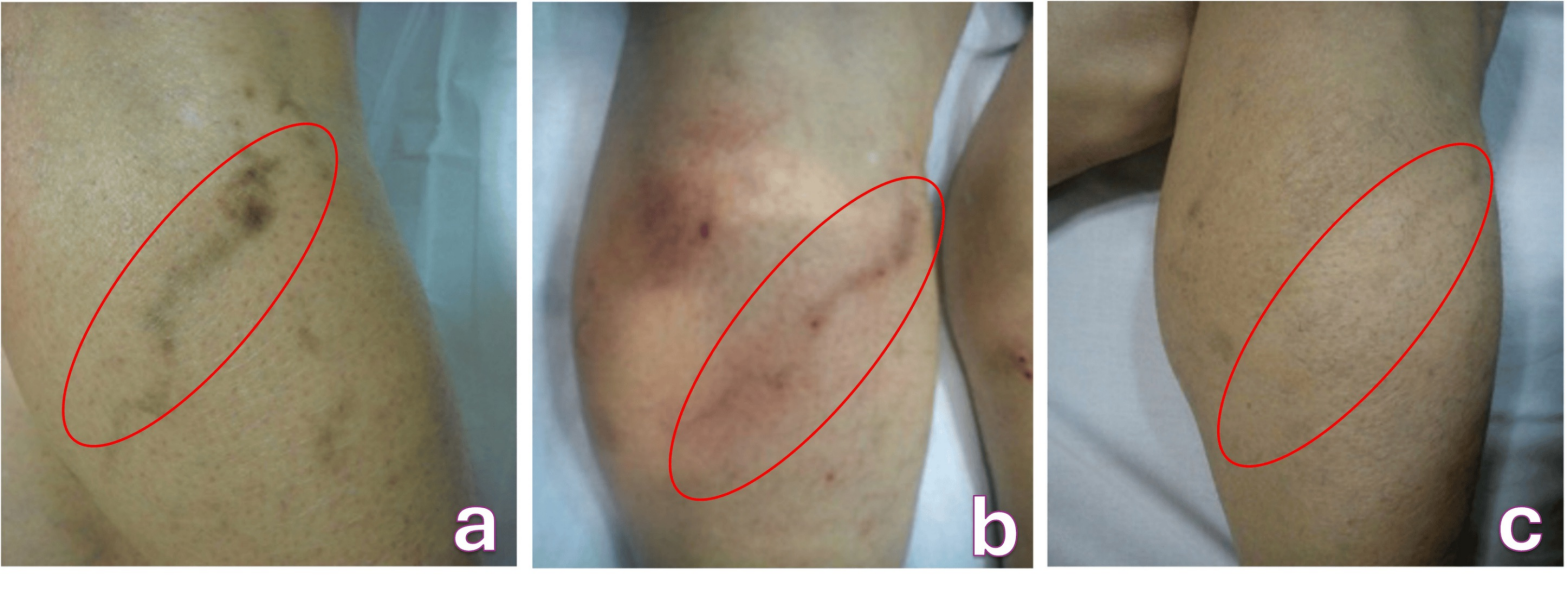
Hyperchromic spot on the course of a calf varicose vein after sclerotherapy with 0.5% polidocanol a. Before treatment, b. Local hyperemia and edema after mesotherapy, c. Final treatment result

In the first case, a 35-year-old woman with Fitzpatrick skin phototype III sought evaluation in May 2023 due to a persistent brownish macule on the medial region of her right calf. The pigmentation had been present for six months and developed shortly after undergoing sclerotherapy with 0.5% polidocanol foam for a reticular vein (Figure 1a). She reported no pain, ulceration, or pruritus but expressed significant cosmetic concern. Her history included melasma and hyperpigmented scars after a cesarean section, both suggestive of a predisposition to pigmentary alterations. She was not using any routine medications.

Physical examination revealed a well-defined, uniform brown macule following the trajectory of the treated vein. There was no palpable cord, edema, or inflammatory change, and the clinical presentation was consistent with post-sclerotherapy hyperpigmentation; therefore, no additional laboratory or imaging tests were deemed necessary.

The patient underwent intradermal mesotherapy with deferoxamine mesylate, delivered through 0.1-0.2 mL injections spaced 1 cm apart across the hyperpigmented region. She experienced only mild and transient erythema and edema that resolved spontaneously within eight hours after each session (Figure 1b). Over the course of treatment, gradual lightening became evident, culminating in complete resolution of the pigmentation after four sessions performed across 16 weeks (Figure 1c).

Case two

**Figure 2:**
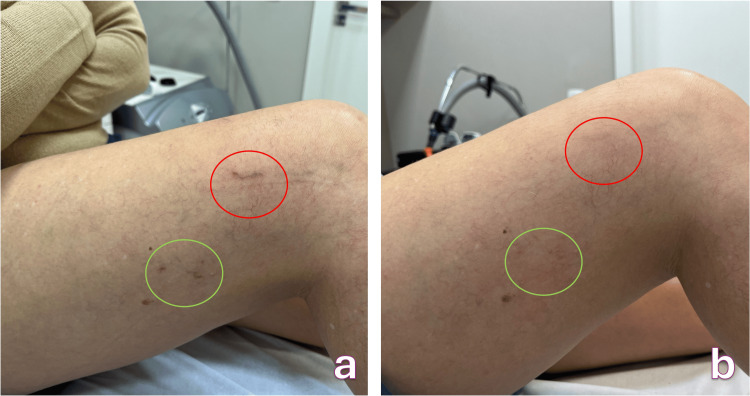
Hyperpigmentation on the lateral side of the right thigh after transdermal laser sclerotherapy a. Before treatment, b. After treatment (telangiectasias were not treated at this time)

In the second case, we have a 58-year-old female patient (Fitzpatrick phototype III) who presented in August 2023 with mild to moderate persistent hyperpigmentation even after four months of sclerotherapy with hypertonic glucose (75%) for telangiectasias on the lateral thigh (Figure 2a).

Her medical history included systemic lupus erythematosus treated with hydroxychloroquine and depression managed with desvenlafaxine. It is worth noting that the use of hydroxychloroquine is also cited as a significant predisposing factor for pigmentary disorders.

The patient tolerated intradermal deferoxamine mesylate well, with no adverse effects observed. Complete clearance of the hyperpigmentation was achieved after three treatment sessions (Figure 2b).

The second patient, a 58-year-old woman with Fitzpatrick phototype III, presented in August 2023 with persistent hyperpigmentation on the lateral aspect of her right thigh (Figure 2a). The pigmentation had developed four months earlier after sclerotherapy for telangiectasias using 75% hypertonic glucose. Her primary complaint was the noticeable darkening of the treated region, although she reported no associated symptoms. Her past medical history included systemic lupus erythematosus treated with hydroxychloroquine (a medication known to contribute to pigmentary disorders) as well as depression managed with desvenlafaxine.

On examination, she exhibited mild to moderate brown macules without associated erythema, texture alteration, or residual telangiectasias. The pattern and chronology were compatible with post-sclerotherapy hyperpigmentation, and no complementary investigations were necessary.

She received monthly intradermal injections of deferoxamine mesylate following the standard protocol. The treatment was well tolerated, with no adverse effects reported throughout the sessions. Progressive improvement was documented in serial photographs, and complete clearance of the hyperpigmented area was achieved after three sessions (Figure 2b).

Case three

**Figure 3:**
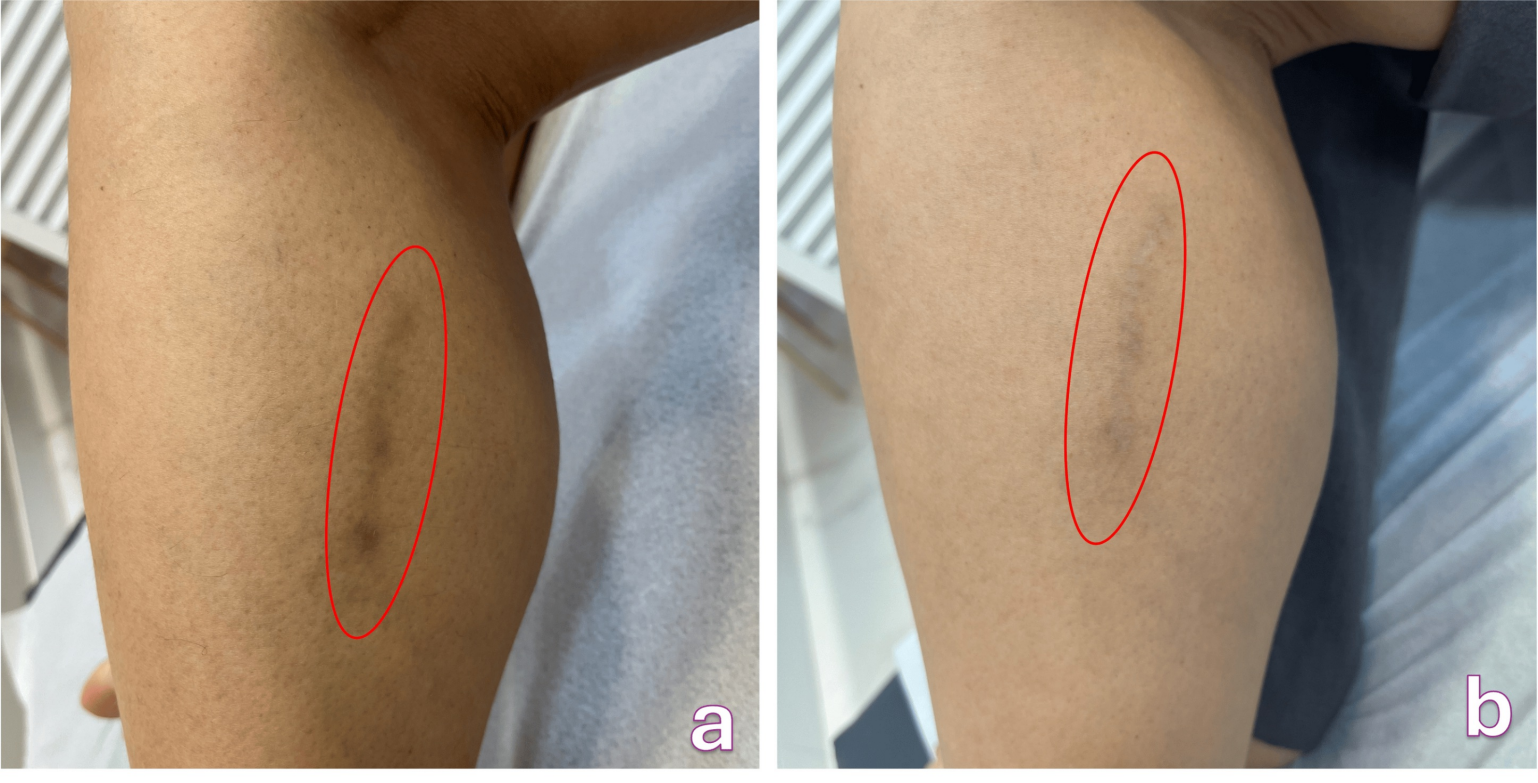
Post-sclerotherapy spot with polidocanol 1% foam on the lateral side of the left calf a. Before treatment, b. After treatment (disappearance of 70% of the stain)

The third case involved a 45-year-old woman with Fitzpatrick phototype IV who presented in January 2024 with a year-long history of dark hyperpigmentation on the lateral region of her left calf. The pigmentation appeared following sclerotherapy using 1% polidocanol foam for varicose veins and had progressively intensified over time, causing considerable aesthetic distress (Figure 3a). Her medical background included the use of combined oral contraceptives and vitamin D supplements, as well as a history of hyperpigmented scars after cosmetic procedures, suggesting increased susceptibility to pigmentary responses.

Clinical examination revealed a large, dark brown macule without erythema, edema, or signs of inflammation. Given the typical appearance and prolonged duration of the lesion, further diagnostic tests were not required.

Monthly mesotherapy sessions with intradermal deferoxamine mesylate were undertaken. After each session, the patient developed mild erythema and slight edema that subsided within two hours. Over the course of three sessions, she achieved approximately 70% improvement in pigmentation, demonstrating partial but significant clinical benefit (Figure 3b). It's important to remember that the treatment endpoint was patient satisfaction.

Among the three patients treated with intradermal deferoxamine mesylate, two achieved complete clearance of hyperpigmentation, while one demonstrated partial clearance (~70%), possibly related to the longer duration of pigmentation (12 months) and the higher concentration of sclerosing agent used in the initial procedure.

The mean time to observable clinical improvement was 13.3 weeks, and all patients reported full satisfaction with their cosmetic results.

No serious or systemic adverse events were reported. Minor, self-limited local reactions included transient burning sensations during injection and mild erythema and edema resolving within eight hours. No cases of hypopigmentation, scarring, infection, or allergic reactions were observed during the treatment course.

## Discussion

This case series demonstrates that focal intradermal administration of deferoxamine mesylate via mesotherapy can accelerate the clinical resolution of residual post-sclerotherapy hyperpigmentation, corroborating the pioneering observations of López et al. [8]. These findings support the pathophysiological hypothesis that the removal or chelation of hemosiderin-derived iron in dermal tissue reduces pigmentation persistence: López et al., in 2001, already demonstrated that subcutaneous administration of deferoxamine significantly accelerates the time to discoloration after sclerotherapy, a report that directly supports the mechanism of action observed in our interventions.

However, there are important differences between studies. While López used weekly subcutaneous injections and reported a rapid reduction in time to depigmentation, our series applied the drug intradermally (mesotherapy) at monthly intervals, with different doses and volumes, and still observed consistent clinical improvement. This technical difference (intradermal vs. subcutaneous; weekly vs. monthly frequency) suggests that more superficial delivery directly into the dermis may be sufficient to achieve a clinical effect, although the small series design does not allow us to conclude which regimen is more effective or faster. Larger, comparative studies would be needed to define the optimal route, dilution, and interval [8].

Furthermore, alternative approaches and preventive strategies against post-sclerotherapy hyperpigmentation have also been studied. Gonzalez Ochoa et al. demonstrated that adjuvant interventions, for example, adding a venoactive agent (sulodexide) to the sclerotherapy protocol, can reduce the occurrence of hyperpigmentation, suggesting that actions that modulate the vascular response and the initial inflammatory process play a role in preventing the problem. Our finding that patients with predisposing factors (history of hyperpigmented scars, chronic use of certain drugs, higher sclerosing agent concentration) showed variable responses is consistent with the literature that relates individual predisposition and procedure characteristics to the probability and severity of pigmentation [5,6].

More recently, randomized intra-individual data comparing sclerotherapy techniques (e.g., foam versus techniques combining cryotherapy/laser) suggest variations in the incidence and intensity of pigmentation according to the technique employed. They demonstrate that techniques with less thermal damage/control of skin trauma can reduce pigmentation intensity, reinforcing the view that both the technical factors of the procedure and post-procedure interventions influence the pigmentary outcome. This complements the observation that intervention with iron chelators may act on one of the final mechanisms of hyperpigmentation (hemosiderin deposition) [9].

From a histopathological perspective, recent studies point to the coexistence of hemosiderin and melanin in post-sclerotherapy lesions, which explains why therapies exclusively focused on removing iron may not produce complete resolution in all cases and why therapeutic combinations (e.g., topical depigmenting agents associated with chelating treatment) make pathophysiological sense. Thus, our results, complete clearing in shorter-duration lesions and partial response in 12-month pigmentation, are consistent with the notion that the older and deeper the pigment deposit, the more difficult complete removal is with iron chelation alone [3,10].

In a complementary way, reviews on post-inflammatory pigmentation emphasize that darker phototypes tend to present more lasting pigmentation and that combined preventive and therapeutic measures (sun protection, depigmenting agents, minimally traumatic treatments) usually offer the best functional and aesthetic results. In this series, two patients had a history of previous skin pigmentation, which may have predisposed them to greater persistence and therapeutic difficulty in one of the cases, as described in the literature [4].

In summary, our findings corroborate previous evidence that deferoxamine can accelerate the resolution of post-sclerotherapy hyperpigmentation and add that the intradermal route (mesotherapy) is a tolerable and potentially effective alternative. However, important limitations remain: very small sample size, absence of a control group, and subjective and photographic assessments without quantitative instrumental measurements. Therefore, randomized studies comparing (a) different routes of administration (intradermal vs. subcutaneous), (b) dose/frequency regimens, (c) combined approaches (chelating agent + depigmenting agent), and (d) prevention with venoactive agents or modified sclerotherapy techniques are needed to establish recommended protocols.

## Conclusions

Mesotherapy with deferoxamine mesylate has been demonstrated to be a safe and effective therapeutic modality for the management of persistent hyperpigmentation following sclerotherapy. Noticeable clinical improvement occurred within a substantially shorter period than the timeframe typically reported for spontaneous resolution, with no clinically significant adverse events observed. These preliminary findings support the therapeutic potential of desferrioxamine mesylate in this context; however, further prospective, controlled studies are warranted to validate these outcomes and to establish standardized clinical protocols for its optimal use.
